# Retrieval of patent ductus arteriosus device embolization using hybrid approach: a case report

**DOI:** 10.1186/s43044-024-00595-y

**Published:** 2024-12-20

**Authors:** Uma Devi Karuru, Sadanand Reddy Tummala, T. Naveen, Sai Kumar Mysore, Kiran Kumar Kanjerla

**Affiliations:** Department of Cardiology, ESIC Medical College and Super Speciality Hospital, Room no 107, 1 st floor, Sanath Nagar, Hyderabad, 500038 India

**Keywords:** Patent ductus arteriosus, Device embolization, Device retrieval

## Abstract

**Background:**

Patent ductus arteriosus (PDA) is a congenital heart defect that requires closure to prevent complications like heart failure and pulmonary hypertension. Catheter-based closure using devices such as the Amplatzer duct occluder is the preferred method due to its minimally invasive nature. However, device embolization is a rare but recognized complication, particularly in small children or high-flow PDAs.

**Case presentation:**

We report a rare and complex case of spontaneous embolization of a PDA closure device into the descending aorta in an 11-month-old female. The patient, with a history of recurrent lower respiratory tract infections and poor weight gain, underwent a PDA closure procedure after a thorough assessment. During the procedure, the Amplatzer Duct Occluder I device unexpectedly migrated into the descending aorta. Despite initial attempts at percutaneous retrieval using a goose neck snare, the device lodged in the left common iliac artery due to size discrepancy. Further snaring was abandoned to prevent the risk of artery dissection. The patient was then taken for emergency surgical exploration. The cardiovascular surgical team successfully retrieved the device through a left supra-inguinal incision, with no complications post-surgery. The patient showed improved limb perfusion and was discharged one week later.

**Conclusions:**

This case underscores the importance of meticulous procedural planning, multidisciplinary collaboration, and adaptive decision-making in managing rare and challenging complications during PDA device closure. The successful outcome, despite the complex nature of the complication, highlights the effectiveness of combining percutaneous and surgical approaches in pediatric cardiology.

**Supplementary Information:**

The online version contains supplementary material available at 10.1186/s43044-024-00595-y.

## Background

Device embolization of the patent ductus arteriosus is a known complication of PDA device closure [1–4]. The symptoms and complexity of the intervention largely depend on the site of embolization. Here, we present a remarkable and rare occurrence encountered in the cardiac catheterization laboratory: spontaneous embolization of a PDA device into the descending aorta. This complication posed a significant challenge during the procedure. Despite the unexpected nature of the event, our team effectively managed the situation using both percutaneous and surgical approaches.

## Case presentation

An 11-month-old female presented to our outpatient department with a history of three episodes of lower respiratory tract infections, poor weight gain, increased precordial activity, and a suck-rest-suck feeding pattern. There was no evidence of cyanosis, clubbing, excessive irritability, loss of consciousness, or seizures.

### Initial assessment

Hemogram, liver function tests, renal function tests, lipid profile, and thyroid function tests were normal. Viral markers turned negative. Electrocardiogram showed sinus rhythm with left atrial and left ventricular enlargement. Chest X-ray revealed mild cardiomegaly with LA and LV enlargement and two-dimensional echocardiography showed small PDA (2.5 mm) with left-to-right shunting, mild pulmonary hypertension, and preserved biventricular function. Given her symptomatic burden, the decision was made to proceed with PDA device closure after obtaining parental consent.

### Procedure

A venous-only approach was employed. The PDA was crossed using a J-tipped 0.035'' Terumo wire over a multipurpose A1 (MPA 1) catheter, which was subsequently exchanged with a Cook Medical Amplatz extra stiff 0.035'' J tip guide wire. Despite resistance in the pulmonary end of the PDA, the 6-F Amplatzer PDA delivery sheath was eventually advanced successfully.

### Complication

Upon deployment of the Amplatzer Duct Occluder I (5 mm × 4 mm) (AGA Medical Corporation, Plymouth, Minnesota, USA), the device appeared to jump toward the aortic end immediately after release. Echocardiography revealed persistence of the PDA. We planned to retrieve the device from the pulmonary side while stabilizing it from the aortic end with a pigtail catheter.

**Fig. 1:**
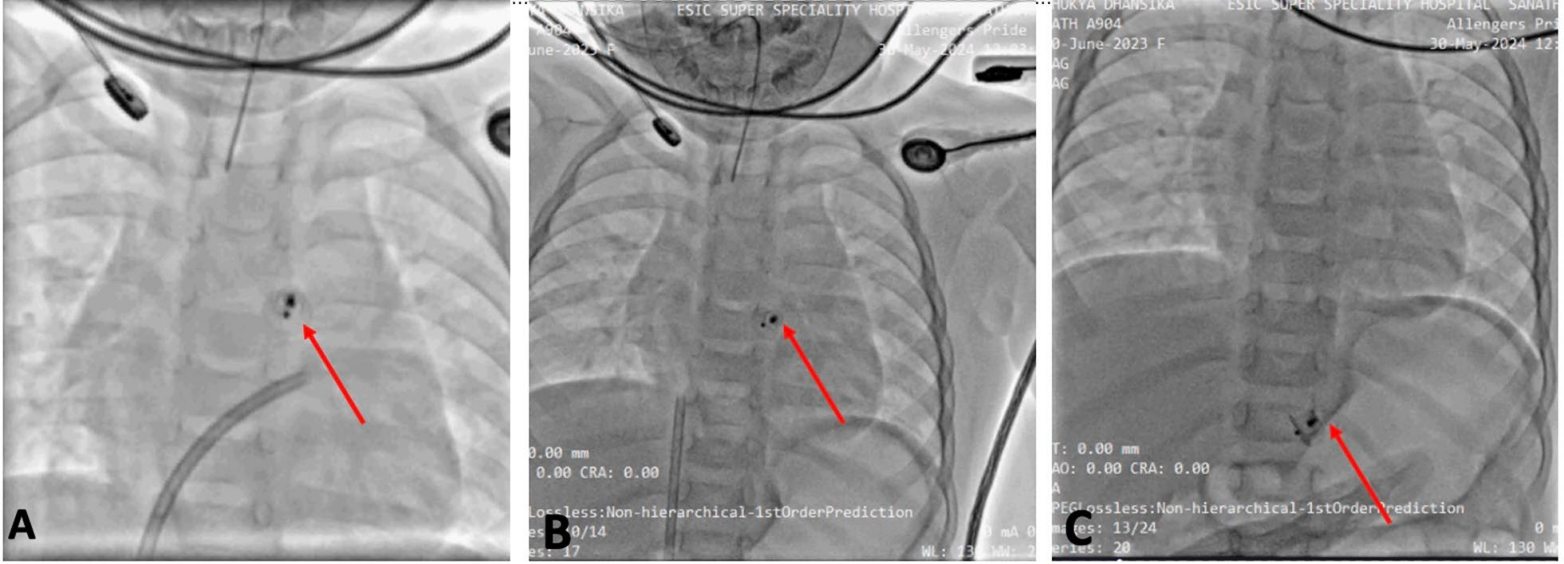
Fluoroscopy- Antero posterior view image depicting the migrated PDA device in the descending aorta below diaphragm. Red arrow showing PDA device near T5-T6 junction **A**, at T7 **B**, at T12 **C**

During arterial access, it was noticed that the device had migrated to the supradiaphragmatic aorta and subsequently to the infradiaphragmatic aorta, just above the origin of the renal arteries, due to high aortic flow (Fig. 1). We decided to retrieve the device from the aortic end with a goose neck snare, aiming to either fully remove it or reposition it to the level of the iliac bifurcation to avoid major abdominal exploration due to inability to pass the guiding catheter into the PDA from the venous end due to ductal spasm (Video S3, Video S4).

### Intervention

The team faced a significant challenge in retrieving the device from the aorta given the small caliber of the vessel, aiming to avoid major exploratory abdominal surgery. After several attempts, the device was retrieved into the left common iliac artery with an Amplatz 10-mm goose neck snare, where it became lodged due to size discrepancy (Video S1). There was tight resistance and automatic jumping of the bulky device just below the iliac bifurcation compared to the left common iliac artery size. A check angiogram at the left common iliac artery revealed the bulky device and tapered end of the vessel below it (Video S2). Further snaring was not possible and was not attempted due to the anticipated risk of left common iliac artery dissection, and the patient was taken for surgical exploration.

### Surgical retrieval

**Fig. 2:**
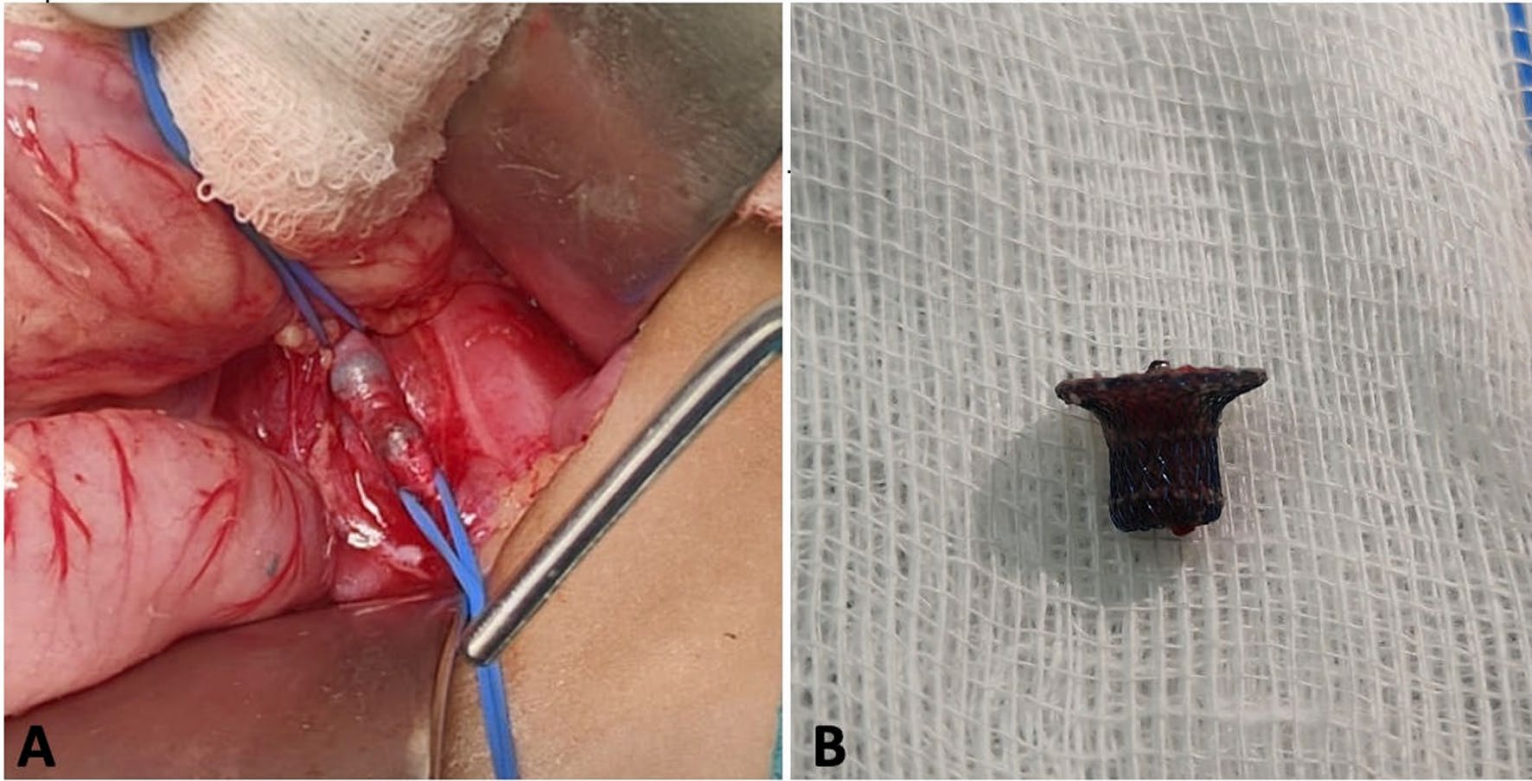
**A** Intra operative image stretching the artery and **B** Post-surgical image showing the removed PDA device

The cardiovascular surgical team prepared for emergency open exploration using left supra-inguinal incision. During surgery, the device was found stretching the vessel with intact intimal layer without dissection and was successfully removed (Fig. 2). Post-surgery, the patient showed improved limb perfusion with no signs of arterial hypoperfusion. She was discharged one week after the procedure.

## Discussion

Patent ductus arteriosus is a congenital heart defect characterized by the persistence of the ductus arteriosus after birth. Closure of the PDA is necessary to prevent complications such as heart failure and pulmonary hypertension. Catheter-based closure using devices like the Amplatzer duct occluder has become the preferred method due to its minimally invasive nature. However, device embolization is a known risk occurring in < 1–5% of cases, particularly in small children or in cases of high-flow PDAs [1–4].

Embolization of the device can occur either into the pulmonary artery or aorta. Aortic embolization is less common than pulmonary artery embolization. Pulmonary artery embolization causes sudden loss of cardiac output and right ventricular strain. Aortic embolization can lead to major organ hypoperfusion and subsequent complications such as bowel gangrene, renal failure, lower limb ischemia, or compartment syndrome [2].

Depending on the site and feasibility of percutaneous retrieval, these events are classified as moderate or major adverse events [5, 6]. Our case highlights a major adverse event requiring surgical intervention. When a device becomes lodged in the ductus, it may sometimes be retrieved by reattaching the delivery cable after advancing a sheath from the venous side. The required snare catheter and sheath sizes depend on the device’s dimensions and location. If the cylindrical body is positioned at the pulmonary end, retrieval may be possible with a sheath just 2-Fr larger than the device delivery size. However, repeated retrieval attempts may cause “infolding” at the sheath tip, obstructing the lumen and hindering device withdrawal.

If the device migrates into the aorta, femoral artery access should be carefully assessed, considering vessel size and pathway before introducing an additional sheath. Bilateral femoral access can facilitate the procedure, though large-diameter sheaths pose a risk of vascular injury, particularly in children. Vessel size may need to be confirmed via ultrasound, and care is essential to avoid aortic wall injury from the device or sheath. If needed, a surgical cutdown or a percutaneous arterial suture device may be employed [7].

In our case, ductal spasm likely caused underestimation of the device size, leading to embolization into the aorta. Since the device is already in the abdominal aorta, retrieval from the pulmonary end would be challenging. Due to body size limitations, introducing a larger sheath into the artery was not feasible, so retrograde retrieval was attempted to safely reposition the device in the abdominal aorta. Percutaneous retrieval distally prevented major abdominal exploratory surgery. We plan to attempt PDA device closure after 6 months with the bigger device, as the child recently underwent open exploratory abdominal surgery.

## Conclusions

The successful management of this PDA device embolization underscores the importance of meticulous procedural planning, skilled execution, and multidisciplinary collaboration in pediatric cardiology. Despite the unexpected complication, the patient recovered well, highlighting the efficacy of combined percutaneous and surgical approaches in managing complex cases.

### Learning objectives

Recognize the potential complications associated with patent ductus arteriosus (PDA) device closure, including device embolization, and differentiate between moderate and major adverse events based on their clinical implications and management strategies. Evaluate the efficacy of percutaneous and surgical approaches in managing rare and complex complications such as PDA device embolization, considering factors such as patient age, device size, anatomical location, and hemodynamic stability. Analyze the importance of multidisciplinary collaboration, procedural planning, and adaptive decision-making in addressing unexpected complications during pediatric cardiology interventions, and formulate strategies to optimize patient outcomes while minimizing procedural risks.

## Supplementary Information

Supplementary Material 1. Video S1. Fluoroscopy-antero-posterior view showing the device retrieval into the left iliac artery.

Supplementary Material 2: Video S2. Fluoroscopy-antero-posterior view showing the device was larger than the left iliac artery.

Supplementary Material 3: Video S3. Two-dimensional echocardiography showing 3-mm patent ductus arteriosus at the level modified aortic valve.

Supplementary Material 4: Video S4. Two-dimensional echocardiography showing 1.5-mm patent ductus arteriosus at the level modified aortic valve when 5-Fr multipurpose catheter was passed across the patent arterial duct and totally occluding the patent ductus arteriosus.

## Data Availability

Not applicable.

## Abbreviations

PDA: Patent ductus arteriosus
ECG: Electrocardiography
CXR: Chest X-ray
LA: Left atrium
LV: Left ventricle
2D ECHO: Two-dimensional echocardiography
